# Acculturative stress, everyday racism, and mental health among a community sample of South Asians in Texas

**DOI:** 10.3389/fpubh.2022.954105

**Published:** 2022-10-24

**Authors:** Shan Mohammed Siddiqui

**Affiliations:** Texas Christian University Fort Worth, Fort Worth, TX, United States

**Keywords:** Asian Americans, racism, acculturation, stress, mental health

## Abstract

South Asian Americans are part of the fastest growing racial/ethnic group in the United States and make up a substantial portion of the U.S. immigrant population. Research on this group has often focused on acculturation, the adoption of different values and behaviors in a new sociocultural environment. While there is evidence to suggest that acculturation (and the stress associated with this process) has a negative effect on the health and well-being of Asian Americans, more recent research has emphasized the need to examine the role of broader social forces—including everyday racism—in impacting mental health. Drawing on the stress process model, this study uses an original survey instrument to investigate the relationships between acculturative stress, anti-Asian racism, and mental health among a community sample of 200 South Asians in Texas. Results from hierarchical multiple regression models indicate that both acculturative stress and everyday racism are strongly linked to higher levels of anxiety-related symptoms and more frequent depressive symptoms. Everyday racism, however, explained variance in these outcomes, well beyond the effect of acculturative stress and other sociodemographic factors. These results underscore the potential benefit and importance of including questions about racism in community health surveys that aim to study health disparities among Asian Americans and highlight the persistence of social issues that U.S. South Asians face.

## Introduction

South Asian Americans are an ethnically, linguistically, and religiously diverse group of people whose ancestry ties back to the southern peninsular region of Asia, sometimes referred to as the Indian subcontinent. Recent trends reveal that South Asians are part of the fastest growing racial/ethnic group in America and—numbering at 5.4 million people—make up a substantial portion of the U.S. immigrant population (1). Although research has shown that immigrants who move to America tend to arrive with better health than native-born residents of similar socioeconomic profiles (2, 3), studies have documented that this “health advantage” erodes over time, possibly due to acculturation in U.S. society (4).

Acculturation refers to the process of minority groups adopting values, beliefs, and behaviors as a result of prolonged contact with the majority group (5). Acculturative stress, then, refers to the tension associated with making these changes and the pressure of adapting to a new socio-cultural environment (6, 7). This stress often includes difficulties with having to learn another language, struggling to balance different social values, and—in the context of this study—negotiating between American and South Asian ways of everyday life.

While there is evidence to suggest that acculturation (and the stress associated with this process) has a strong, negative effect on the health and well-being of foreign-born groups (8, 9), more recent research has emphasized the need to examine the role of *broader* social forces in impacting immigrants' health (10). Some scholars, for example, have argued that poor health among U.S. immigrants may have more to do with structural factors that disadvantage and increase the vulnerability of these communities (11, 12). One such factor is racism.

Operating at multiple levels, racism works to disempower and discriminate against people based on their racial or ethnic background and has been conceptualized as a key source of stress for those who are targeted (13). Multiple studies have shown that experiences with racism have harmful effects on the mental health of racial/ethnic minority groups and are related to participation in unhealthy behaviors as a way of coping with such encounters (14, 15). Although South Asians are viewed by some as being a “model minority” group and often rendered invisible in research on racism, they still face mistreatment based on their race or ethnicity (16, 17) and in some ways are hyper-visible targets. In 2017, for example, the non-profit group South Asian Americans Leading Together (SAALT) documented over 300 bias-related incidents against South Asians and Middle Easterners of multiple faiths (18). This number captured experiences of verbal abuse, harassment, and violence, and represented a 64% increase in bias-related incidents from the previous year. Some scholars have attributed this increase to heightened levels of Islamophobia in the U.S. (19), which have subjected South Asians—who are often perceived as being Muslim based on their physical appearance– to both surveillance and subjugation (20).

Given these occurrences, the recent rise in anti-Asian hate crimes (21), and immigration enforcement policies that have targeted foreign-born people who are studying in the U.S. (22), it is possible that both acculturative stress and racism may be linked to poor mental health among this rapidly-growing and increasingly stigmatized social group. This study, accordingly, examines the links between acculturative stress, everyday racism, and mental health among South Asians in the U.S.—contributing to both the race and immigrant health literatures—and sheds light on how various forms of social stress impact mental health.

Mental health refers to emotional, psychological, and social well-being and reflects the equilibrium between individuals and the environment in which they live (23). Though this concept has garnered more attention in social-scientific research in recent decades, few studies have examined the relationship of mental health to acculturative stress and racism experienced among South Asian Americans. Prior research examining these stressors in *other* groups has found that racism is associated with heightened acculturative stress (24) and that these socio-cultural adversities are linked to worse mental health outcomes, including increased depressive symptoms and greater psychological distress (25, 26). To understand how stress from acculturation and everyday racism might impact the mental health of South Asians in the U.S., I draw on the stress process model.

The stress process model provides a useful theoretical framework for understanding mental health disparities. In this model, exposure to stress is linked to worse mental health, can manifest across different social contexts, and is related to one's status in society (27). In other words, this model suggests that health issues are not randomly distributed in the population but are rather a reflection of social and structural arrangements that systematically disadvantage racial/ethnic minority groups in the U.S. Because South Asians navigate their lives being seen by others as perpetual foreigners (28), some stressors, such as acculturation, may be more salient and associated with poor mental health. For example, prior research has shown that South Asian American adolescents experience stress from having to navigate multiple cultural contexts, which may inhibit their psychological well-being (29). Additionally, because of how South Asians are racialized in the U.S. (and vilified by actors in both the private and public spheres), it is plausible that racism is also linked to negative outcomes and may impact mental health, even after accounting for acculturative stress. By investigating the experiences of this population through a more nuanced lens, this study extends the work of previous scholars by examining how acculturative stress and everyday racism are tied to mental health among U.S. South Asians. Following the stress process model and building upon findings from previous research, this investigation addresses the following questions:

1. Is acculturative stress associated with the mental health of South Asian Americans?
2. Is everyday racism associated with the mental health of South Asian Americans?
3. Does everyday racism explain variance in mental health, beyond the effect of acculturative stress and other sociodemographic factors?

## Methods

After receiving approval from the Institutional Review Board, participants were recruited from the South Asian population in two cities in Texas. This southwestern state is home to one of the largest South Asian populations in the U.S. (30) and was also ranked #4 among states with the most anti-Asian incidents in the past year (31). Recruitment efforts focused on South Asian social networks (on WhatsApp, Facebook, and Twitter), university organizations geared toward South Asian members, houses of worship, and South Asian cultural centers. Similar to previous research on South Asian doctors' experiences with racism in the Texas medical field (32), this investigation invited community members living in the Austin and Houston metropolitan areas to take part in a study about their social experiences living as South Asians in the U.S.

Identifying as South Asian, myself, I recognize that my identity and experiences had some influence on what questions I chose to incorporate in my survey instrument, as well as on the recruitment process. For example, having negative encounters at the airport in the past (and knowing that these interactions happen to other South Asian people) informed my decision to include an item on the discrimination scale about mistreatment while traveling. Additionally, being a member of the target study population, I was able to use my position to gain access to South Asian participants, who may have otherwise been hesitant to participate in the study.

The sampling criteria included: having a South Asian / Desi background, being 18 years of age or older, having the ability to read in English, and residing in the state of Texas. Since research has shown that acculturation impacts South Asians across generations and in various ways (33), both South Asian immigrants and children of immigrants were eligible to participate. After obtaining informed consent, participants completed an online Qualtrics survey. Although it may have also been helpful to distribute the survey in person, I was unable to do so because of the COVID-19 pandemic. This is a limitation that I acknowledge and expand upon later.

Data were collected from June 2021 to October 2021. The analytic sample consisted of 200 participants. The sample size was determined by the generally accepted rule of N ≥ 50 + 8 m, where m is the number of independent variables (34). Since there were 13 independent variables in the study, a minimum of 154 cases (50 + 8 × 12 = 154) were needed for a regression analysis.

### Measures

#### Dependent variables

The main dependent variable for this investigation included two measures of mental health: depressive symptoms and anxiety-related symptoms. The measure of depressive symptoms was adapted from the Center for Epidemiologic Studies Depression Short Form (CES-D-10), which is a 10-item scale made for use with non-clinical samples, and assesses the frequency of current depressive symptoms (35). All items were incorporated into the survey, including: being bothered by things that usually are not bothersome, having trouble keeping one's mind on what they are doing, sleeping restlessly, having poor appetite, feeling sad, feeling lonely, feeling depressed, feeling like everything took a lot of effort, feeling happy, and enjoying life. This scale measured depressive symptoms on a Likert-type scale (*1* = *never, 4* = *most or all of the time*) and coded (or reverse-coded) items so that higher scores reflected higher frequency of depressive symptoms. Cronbach's alpha was 0.90, indicating high statistical reliability.

The measure of anxiety-related symptoms was adapted from the short-form of the State Trait Anxiety Inventory (STAI) developed by Spielberger (36), which is a reliable and valid form (37) consisting of 6 items that assesses the presence and absence of anxiety-related symptoms, including feeling: anxious, worried, nervous, comfortable, pleasant, and at ease. Participants were asked about how they often they felt certain emotions and responded to each of these items with “not at all” (coded as 1), “a little” (coded as 2), “somewhat” (coded as 3), or “very much so” (coded as 4). Items were coded (or reverse-coded) so that higher scores reflected higher levels of anxiety. Cronbach's alpha was 0.87, indicating high statistical reliability.

#### Independent variables

A key independent variable in this study was acculturative stress. Similar to previous research on acculturation, discrimination, and mental health among Asian populations (25), this variable was adapted from the National Latino and Asian American Study (NLAAS) and consisted of 7 items to which participants could respond “yes” or “no.” The 7 items included: feeling guilty for leaving or being separated from family overseas, having difficulties interacting with others because of English nonproficiency, being treated badly because of speaking another language or speaking English with a South Asian accent, being concerned or questioned by others about one's legal status in the U.S., being concerned about running into trouble with immigration officials if one were to go to a government agency, avoiding seeking out healthcare due to fear of immigration officials, and whether life has become more difficult to navigate as an immigrant or child of immigrants in the U.S. A factor analysis was performed to examine the underlying structure of acculturative stress. All items except “avoiding seeking out healthcare due to fear of immigration officials” mapped on well to the scale. Scores ranged from 0 to 6, with higher scores reflecting higher levels of acculturative stress. Cronbach's alpha was 0.62, indicating moderately high statistical reliability.

Participants were also asked about experiences with everyday racism through the Everyday Discrimination Scale (38), a reliable and valid scale for assessing routine experiences with unequal treatment (39). Since this study focused on discrimination based on race/ethnicity, the items were modified to specifically account for events that occurred due to being South Asian. These items included: being treated with less courtesy, being treated with less respect, receiving poorer service at restaurants or stores, having people acting as if they are afraid of you, having people acting as if they are better than you, being called names or insults, and being physically threatened or harassed. Based on research that shows that South Asian Americans, in particular, get treated differently when traveling (40) and seeking out social relationships online (41), I also added the following 3 items to the scale: being treated with less courtesy when traveling, having people ignore you in online forms of communication, and having people act as if they are disgusted by you. These items are unique in that they may be more salient for South Asians because of the way they are racialized in the U.S. Similar to acculturative stress, I performed a factor analysis for this variable. All items were used to develop the construct, which measured everyday racism on a Likert-type scale (1 = never, 6 = almost every day), with higher scores indicating higher levels of racism. Cronbach's alpha was 0.89, indicating high statistical reliability.

#### Sociodemographic variables

The sociodemographic variables in this study included: age, gender, ethnicity (Indian, Pakistani, Bangladeshi, or other), immigrant legal status (U.S. citizen, lawful permanent resident, or other, which included nonimmigrants on a temporary visa, undocumented immigrants, and refugees), educational attainment, English language proficiency (excellent, good, or fair), marital status, income, religious identity (Muslim, Hindu, Christian, or other), contact with a mental health therapist, and years lived in the U.S. These variables have been used in previous studies that examined the relationship between acculturative stress, discrimination, and mental health among communities of color (24, 25).

### Analytic strategy

Stata/SE 16 was used to analyze data from the online survey. First, a descriptive analysis of sociodemographic variables was conducted and is presented in Table 1. Next, I examined the response distribution for each item on the scales, as well as bivariate analyses on the links between mental health symptoms and key independent variables, which can be found in the Supplementary materials. Finally, hierarchical multiple regression models were estimated to examine the relationships between acculturative stress, everyday racism, and mental health among South Asian respondents and presented in Tables 2, 3. This technique is useful for comparing different statistical models and can demonstrate if certain predictors explain a significant amount of variance in the outcome(s) of interest after accounting for all other variables (42). One strength of this approach is that the researcher can select the order in which the variables are entered, based on a theoretical rationale and/or their research questions (43). Hierarchical multiple regression models have been used in other studies that examine acculturation, discrimination, and/or mental health (25, 44, 45)—and in the context of this investigation—can reveal if everyday racism explains variance in depressive or anxiety-related symptoms, above and beyond the effect of acculturative stress.

**Table 1:** Descriptive statistics.

**Variables**	**% of sample**
**Age**
18–25 years	19.29
26–34 years	52.14
35–44 years	14.29
45–54 years	7.14
55 years or older	7.15
**Gender**
Male	31.42
Female	68.57
**Education**
High school or less	2.86
Some college/Associate's degree	9.57
Bachelor's degree	31.43
Graduate degree	56.43
**Income**
$0–$24,999	8.43
$25,000–$49,999	11.57
$50,000–$74,999	15.71
$75,000–$99,999	12.14
$100,000+	53.57
**Ethnicity**
Bangladeshi	13.44
Indian	36.82
Pakistani	49.75
**English proficiency**
Fair or good	13.43
Excellent	86.43
**Religious identity**
Muslim	72.59
Christian	9.64
Hindu	8.63
Other	9.14
**Legal status**
U.S. citizen	83.42
Lawful permanent resident	6.22
Other	10.36
**Seeing a therapist**
No	96.63
Yes	3.37
	**Mean (SD)**
**Years in the U.S**.	24.52 (9.28)
**Key independent variables**
Acculturative stress	0.2471 (0.2333)
Everyday racism	0.3218 (0.1789)
**Dependent variables**
Anxiety-related symptoms	0.3685 (0.2376)
Depressive symptoms	0.2946 (0.2443)

**Table 2:** Hierarchical multiple regression models predicting anxiety-related symptoms (*n* = 200).

**Independent variables**	**Model 1**		**Model 2**		**Model 3**	
	**β**	**SE**	**β**	**SE**	**β**	**SE**
**Age (ref: 18–15 years)**						
26–34 years	−0.0477	0.0575	−0.0315	0.0562	−0.0220	0.0553
35–44 years	−0.0869	0.0750	−0.0677	0.0733	−0.0621	0.0721
45–54 years	−0.2025*	0.0810	−0.1815*	0.0792	−0.1726*	0.0779
55 years or older	−0.2193*	0.0963	−0.1920*	0.0942	−0.1680	0.0940
**Gender (ref: Male)**						
Female	0.0291	0.0396	0.0382	0.0388	0.0395	0.0381
**Education (ref: Graduate degree)**						
High school or less	−0.0761	0.0940	−0.0844	0.0916	−0.0745	0.0902
Some college/Associate's degree	0.0162	0.0722	−0.0097	0.0704	−0.0063	0.0691
Bachelor's degree	−0.0207	0.0431	0.0037	0.0426	0.0073	0.0421
**Income (ref: $100,000+)**						
$0–$24,999	0.0601	0.0720	0.0684	0.0710	0.0891	0.0702
$25,000–$49,999	0.0353	0.0762	0.0498	0.0747	0.0686	0.0738
$50,000–$74,999	0.0142	0.0526	−0.0092	0.0518	−0.0004	0.0511
$75,000–$99,999	0.0117	0.0535	0.0138	0.0523	0.0201	0.0517
**Years in the U.S**.	0.0010	0.0028	0.0002	0.0027	−0.0006	0.0027
**Ethnicity (ref: Pakistani)**						
Bangladeshi	0.0241	0.0664	−0.0030	0.0659	−0.0165	0.0680
Indian	0.1472**	0.0526	0.1208*	0.0520	0.1184*	0.0510
**English proficiency (ref: Excellent)**						
Fair or good	−0.0315	0.0532	−0.0518	0.0522	−0.0605	0.0515
**Religious identity (ref: Muslim)**						
Christian Hindu	−0.0861 −0.1671*	0.0806 0.0770	−0.0490 −0.1493*	0.0794 0.0752	−0.0337 −0.1309	0.0781 0.0744
Other	−0.1832*	0.0710	−0.1744*	0.0692	−0.1719*	0.0683
**Legal Status (ref: U.S.-born Citizen)**						
Lawful permanent resident	−0.0422	0.0845	−0.0869	0.0843	−0.0569	0.0839
Other non-U.S. citizen	−0.0165	0.0823	−0.0818	0.0829	−0.0624	0.0827
**Seeing Therapist (ref: No)**						
Yes	0.0476	0.0604	0.0516	0.0589	0.0559	0.0578
**Acculturative stress**			0.2196**	0.0792	0.1215	0.0877
**Everyday racism**					0.2750*	0.1069
*R* ^2^	0.193		0.239*		0.276**	
*ΔR^2^* from previous model			0.046*		0.037**	

* p < 0.05, **p < 0.01.

**Table 3:** Hierarchical multiple regression models predicting depressive symptoms (*n* = 200).

**Independent variables**	**Model 1**		**Model 2**		**Model 3**	
	**β**	**SE**	**β**	**SE**	**β**	**SE**
**Age (ref: 18–15 years)**						
26–34 years	−0.0015	0.0594	0.0090	0.0592	0.0216	0.0570
35–44 years	−0.0381	0.0775	−0.0230	0.0774	−0.0146	0.0741
45–54 years	−0.1407	0.0839	−0.1274	0.0836	−0.1153	0.0802
55 years or older	−0.1913	0.0987	−0.1709	0.0986	−0.1368	0.0958
Gender (ref: Male)
Female	0.0384	0.0410	0.0431	0.0409	0.0450	0.0392
**Education (ref: Graduate degree)**						
High school or less	−0.0073	0.0976	−0.0146	0.0970	0.0000	0.0932
Some college/Associate's degree	0.0598	0.0759	0.0628	0.0754	0.0678	0.0722
Bachelor's degree	−0.0229	0.0443	−0.0082	0.0445	−0.0034	0.0429
**Income (ref: $100,000+)**						
$0–$24,999	0.0380	0.0749	0.0411	0.0753	0.0718	0.0726
$25,000–$49,999	0.0619	0.0809	0.0702	0.0808	0.0971	0.0779
$50,000–$74,999	0.0311	0.0548	0.0167	0.0550	0.0298	0.0529
$75,000–$99,999	0.0521	0.0546	0.0542	0.0546	0.0649	0.0526
**Years in the U.S**.	0.0002	0.0029	−0.0003	0.0029	−0.0015	0.0028
**Ethnicity (ref: Pakistani)**						
Bangladeshi	0.1507*	0.0689	0.1332	0.0698	0.1100	0.0704
Indian	0.1606**	0.0545	0.1417*	0.0550	0.1379*	0.0527
**English proficiency (ref: Excellent)**						
Fair or good	0.0179	0.0542	0.0053	0.0543	−0.0067	0.0522
**Religious identity (ref: Muslim)**						
Christian Hindu	−0.0683 −0.1711*	0.0838 0.0812	−0.0414 −0.1626*	0.0842 0.0808	−0.0198 −0.1360	0.0809 0.0780
Other	−0.0927	0.0725	−0.0844	0.0721	−0.0833	0.0694
**Legal Status (ref: U.S.-born Citizen)**						
Lawful permanent resident	−0.0365	0.0881	−0.0685	0.0895	−0.0246	0.0869
Other non-U.S. citizen	−0.0933	0.0829	−0.1335	0.0850	−0.1028	0.0829
**Seeing Therapist (ref: No)**						
Yes	0.1120	0.0616	0.1178*	0.0570	0.1229*	0.0586
**Acculturative stress**			0.1545*	0.0835	0.0109	0.0902
**Everyday racism**					0.4033***	0.1106
*R* ^2^	0.219		0.239*		0.309**	
*ΔR^2^* from previous model			0.020*		0.070**	

*p < 0.05, **p < 0.01, ***p < 0.001.

## Results

Table 1 shows the distributions of sociodemographic factors among the community sample of South Asians in Texas.

According to Table 1, the majority of the respondents (52.14%) fell between the ages of 26 and 34 years old. Most respondents (68.57%) also identified as female. Regarding educational attainment, 2.86% had a high school diploma, 7.86% finished some college, 31.43% had a bachelor's degree, and 56.43% completed a graduate or advanced degree. With respect to income, 15% of the sample earned $49,999 or less. 53.57%, on the other end, earned over $100,000 per year. 86.43% of the sample had excellent proficiency in English, while 11.43% reported fair or good proficiency. Regarding marital status, 52.14% of the sample was married, 3.57% was divorced or separated, and 44.29% had never married. The overwhelming majority of the sample (83.42%) identified as U.S. citizens. 6.22% were lawful permanent residents, and 10.36% had another legal status. When asked about contact with a mental health provider, only 10.95% of respondents were seeing a therapist, while 89.05% were not. The mean scores of acculturative stress and everyday racism were 0.2471 (SD = 0.2333) and 0.3218 (SD = 0.1789), respectively. Interestingly, there were two items on the everyday racism scale that occurred “often” by about one in five participants. One of these items was being treated with less courtesy while traveling (17.30%). Findings from the hierarchical multiple regression models examining acculturative stress, everyday racism, and anxiety-related symptoms are reported below in Table 2.

Table 2 shows results from hierarchical multiple regression models predicting anxiety-related symptoms among South Asian respondents. The results display regression coefficients (β), which represent the mean change in anxiety-related symptoms for one unit of change in the predictor variable, holding all other variables constant. When estimating hierarchical multiple regression models, the variables are entered in distinct blocks or steps (46). In the initial model (“Step 1”), I included sociodemographic control variables only. In the second model (“Step 2”), I introduced acculturative stress. Then, in the last model (“Step 3”), I added everyday racism to see if this variable played a significant contribution in explaining additional variance in mental health, well beyond what was already included in the previous models.

Results from Step 2 showed that for each unit increase in acculturative stress, a 0.2196 unit increase in anxiety-related symptoms was predicted, holding all other variables constant (*p* < 0.01). R^2^ increased significantly from 0.193 to 0.239 (*p* < 0.05), suggesting that acculturative stress played an important role in explaining variance in anxiety. Furthermore, in Step 3, results showed that for each unit increase in everyday racism, a 0.2750 unit increase in anxiety-related symptoms was predicted (*p* < 0.05). When everyday racism was added to the model, the effect of acculturative stress on anxiety-related symptoms also diminished. In this final step, R^2^ increased significantly from 0.239 in Model 2 to 0.276 in Model 3 (*p* < 0.01), highlighting the unique contribution of everyday racism in explaining variance in anxiety-related symptoms, even beyond the effect of acculturative stress. A similar step-wise process was conducted in Table 3, which examined acculturative stress, everyday racism, and depressive symptoms among South Asian respondents.

Step 1 in Table 3 included sociodemographic controls. Acculturative stress was added in Step 2. In this step, results showed that for each unit increase in acculturative stress, a 0.1545 unit increase in depressive symptoms was predicted, holding all other variables constant (*p* < 0.05). R^2^ increased significantly from 0.219 to 0.239 (*p* < 0.05), suggesting that acculturative stress played an important role in explaining variance in depression. Lastly, in Step 3, everyday racism was incorporated. Results showed that for each unit increase in everyday racism, a 0.4033 unit increase in depressive symptoms was predicted (*p* < 0.001). Similar to before, when racism was added to the model, the effect of acculturative stress diminished. In this final step, R^2^ increased significantly from 0.239 to 0.309 (*p* < 0.001), once again underscoring the unique contribution of everyday racism in explaining variance in this second measure of mental health.

## Discussion

This study, to my knowledge, is one of the first to examine various measures of mental health among South Asian Americans and their relation to both acculturative stress and everyday racism. Although a large body of work has examined the role of acculturation in contributing to health issues among immigrants (47–49), limited research has investigated this process (in conjunction with racism) among this increasingly racialized and rapidly-growing group. Studying both factors is important for understanding how multiple stressors can impact immigrant communities as they navigate various socio-cultural contexts. As a result, this investigation contributes to the literature by advancing knowledge on South Asian Americans' social experiences (including perceptions of anti-Asian racism) and their relation to mental health.

Prior studies have shown that acculturative stress is linked to mental health issues among Latin American immigrants in international contexts (50), as well as other Asian populations in the U.S. (51–53). Scholars have also found that discrimination is associated with worse emotional well-being among Gujarati Americans (54) and depression among U.S. South Asians more generally (55). In line with this research, I found that acculturative stress and everyday racism were strongly linked to higher levels of anxiety-related symptoms and more frequent depressive symptoms among South Asians in Texas. Importantly, however, everyday racism explained variance in these mental health outcomes, well beyond the effect of acculturative stress. This result is congruent with existing literature, which shows that while acculturation and discrimination both have a negative impact on health, discrimination can have a unique and relatively stronger effect (25, 56).

My findings underscore the potential benefit and importance of including questions about racism in local health surveys (which are often missing), especially those targeted toward communities of color. For example, one of the unique characteristics about the survey used in this study is that it contained specific questions about racism that may be more salient for South Asians. As the results from supplementary analyses showed, some of these encounters (e.g., experiencing discrimination while traveling) occurred often in almost a fifth of the sample. Theoretically, the results also challenge the model minority myth by highlighting the persistence of social issues that South Asians experience, which can serve as a major source of stress and contribute to health disparities.

These findings, however, should be interpreted in light of several limitations. First, participants were recruited from only two cities in Texas. It is possible that because South Asians may be more concentrated in large urban areas, their social experiences may differ from those who live in smaller rural towns. This study, however, was not able to take into account geographic variation across settings. Second, responses were based on self-report measures that relied on retrospective collections of particular social encounters. As a result, it is possible that respondents may underestimate or overestimate their experiences with everyday racism. Third, the study's survey was administered in English, and due to the COVID-19 pandemic, its format was online-only. Consequently, I was not able to reach participants who may not be able to read in English or do not have regular access to the Internet. This gap may have impacted the findings, especially if South Asians who did not receive formal education in English and/or come from low-income backgrounds may be impacted more by acculturative stress and everyday racism but were not well-represented in the study. Fourth, while certain kinds of social/professional support were included in the survey, it is possible that there are other important factors, such as community networks (and ties within these networks), that were not incorporated but may also be relevant to mental health. Fifth, results reported from the models were associations, so I am not able to make causal claims using this data. To understand causal relationships, a different type of dataset and analytic strategy would be needed. Lastly, due to the small, majority-Pakistani, and majority-female sample, findings may not be generalizable to the South Asian population in the U.S., which is majority-Indian and consists of slightly more men than women (57).

These limitations point to several avenues for future research. First, studies might examine South Asians' experiences with acculturation and racism in different parts of America, which may yield different outcomes for mental health, depending on this group's social and neighborhood contexts. Second, future research may consider studying the social experiences of South Asians using longitudinal data, such as through daily diaries, which may highlight the effects of racism in real-time and capture any period effects where insults to mental health may be more pronounced. Third, capturing attributions to discrimination would provide more data on why people believe they are being treated unequally. This information would be useful for studying South Asian participants, who may be targeted for reasons beyond race/ethnicity, such as their perceived faith. Fourth, translating surveys into multiple South Asian languages and making the surveys available to complete in person as well as online would have greater reach and could potentially recruit more participants. With these larger sample sizes, studies could investigate how various structures of inequality (e.g., racism, sexism, xenophobia, etc.) intersect to shape health outcomes among South Asians on a broader scale. Lastly, future research might consider studying South Asian Americans in comparison to other immigrant groups, such as communities from Latin America. Doing so may provide empirical insights on how the social experiences of one racialized group shape (and are shaped by) the experiences of another.

In conclusion, this investigation enhances knowledge on the relationship between acculturative stress, everyday racism, and mental health, using a community sample of South Asians in Texas. The results of the study yield multiple implications. First, the findings suggest that when researching immigrant health, it is important to focus not only on acculturation, but to also consider the impact of broader social forces, such as racism. Studying acculturation, alone—which places the responsibility for poor mental health on immigrants—can lead to deficit perspectives (e.g., thinking that immigrants experience negative outcomes because they “lack” the ability to integrate). By shifting attention to more external factors and acknowledging the potential harm that discrimination can cause, it may be possible to identify tactics, such as anti-racist policies, that can reduce discrimination and/or mitigate its effect on health. Knowledge of these strategies could be relevant to stakeholders and policymakers who are working to address acts of xenophobia and anti-Asian racism on a local level. The results also yield implications for mental health providers to address the psychological needs of patients from this community, which may parallel the needs of those from other immigrant groups. Being familiar with the impact of acculturative stress and everyday racism, for example, may help providers identify interventions that can help individuals cope with these problems. Before that, however, scholars may need to continue disaggregating data on Asian Americans to uncover different experiences within this diverse population and determine which groups may be disproportionately impacted by various health issues. By the studying the social experiences of South Asians and other marginalized groups through a more nuanced lens, public health officials, researchers, and practitioners will have a better understanding of how various forms of social stress impact minority health.

## Data availability statement

The raw data supporting the conclusions of this article will be made available by the authors, without undue reservation.

## Ethics statement

The studies involving human participants were reviewed and approved by University of Texas at Austin Institutional Review Board. The participants provided their written informed consent to participate in this study.

## Author contributions

The author confirms being the sole contributor of this work and has approved it for publication.

## Funding

The author was granted funds from Texas Christian University's Open Access Fund.

## Conflict of interest

The author declares that the research was conducted in the absence of any commercial or financial relationships that could be construed as a potential conflict of interest.

## Publisher's note

All claims expressed in this article are solely those of the authors and do not necessarily represent those of their affiliated organizations, or those of the publisher, the editors and the reviewers. Any product that may be evaluated in this article, or claim that may be made by its manufacturer, is not guaranteed or endorsed by the publisher.
