# Supplementary material for: Acculturative stress, everyday racism, and mental health among a community sample of South Asians in Texas
Source: Front Public Health. 2022 Oct 24;10:954105. doi: 10.3389/fpubh.2022.954105 (PMC9638105; doi:10.3389/fpubh.2022.954105)
Supplement: Supplementary file 1 [file Table_1.docx]

**Supplementary Materials**

**Appendix Table 1 – Response Distribution for Acculturative Stress**

| Item | Yes (%) | No (%) |
| --- | --- | --- |
| Do you ever feel guilty for being separated from loved ones who are abroad or leaving family overseas? | 33.85 | 66.15 |
| Do you ever have difficulties interacting with others because of English proficiency? | 6.77 | 93.23 |
| Do you ever get treated badly because of speaking another language around others or speaking English with an accent? | 16.15 | 83.85 |
| Do you ever get concerned about your legal status in the U.S.? | 11.98 | 88.02 |
| Regardless of your legal status, do you ever get worried about being approached by immigration officials if you go to the airport or a government agency? | 48.96 | 51.04 |
| In recent years, has life become more difficult to navigate as an immigrant or child of immigrants in the U.S.? | 38.22 | 61.78 |
