# Supplementary material for: Acculturative stress, everyday racism, and mental health among a community sample of South Asians in Texas
Source: Front Public Health. 2022 Oct 24;10:954105. doi: 10.3389/fpubh.2022.954105 (PMC9638105; doi:10.3389/fpubh.2022.954105)
Supplement: Supplementary file 2 [file Table_2.docx]

**Supplementary Materials**

**Appendix Table 2 – Response Distribution for Everyday Racism**

| Item | Never % | Rarely % | Sometimes % | Often % | Always % |
| --- | --- | --- | --- | --- | --- |
| Been treated with less courtesy than people of other racial/ethnic backgrounds? | 14.59 | 27.03 | 47.03 | 10.81 | 0.54 |
| Been treated with less respect than people of other racial/ethnic backgrounds? | 13.51 | 31.35 | 47.03 | 7.57 | 0.54 |
| Received worse service than people of other racial/ethnic backgrounds in restaurants or stores? | 17.93 | 34.78 | 39.67 | 5.98 | 1.63 |
| Had people act as if they are afraid of you? | 45.41 | 25.95 | 25.41 | 3.24 | 0.00 |
| Had people act as if they are better than you? | 16.22 | 19.46 | 38.92 | 23.78 | 1.62 |
| Been called offensive names or insults? | 35.14 | 38.38 | 22.70 | 2.70 | 1.08 |
| Been physically harassed or threatened? | 70.27 | 21.08 | 7.57 | 1.08 | 0.00 |
| Been treated with less courtesy when traveling (e.g., at airports)? | 25.41 | 24.32 | 28.11 | 17.30 | 4.86 |
| Had people ignore you online (e.g., in emails or on dating websites)? | 50.54 | 22.28 | 16.30 | 8.15 | 2.72 |
| Had people act as if they are disgusted by your presence? | 58.38 | 28.11 | 11.35 | 1.62 | 0.54 |
