# Supplementary material for: Acculturative stress, everyday racism, and mental health among a community sample of South Asians in Texas
Source: Front Public Health. 2022 Oct 24;10:954105. doi: 10.3389/fpubh.2022.954105 (PMC9638105; doi:10.3389/fpubh.2022.954105)
Supplement: Supplementary file 3 [file Table_3.docx]

**Supplementary Materials**

**Appendix Table 3 – Pearson Correlations**

| **Variables** | **1** | **2** | **3** | **4** | **5** |
| --- | --- | --- | --- | --- | --- |
| Years in the U.S. | 1.0000 |  |  |  |  |
| Acculturative stress | -0.1624* | 1.0000 |  |  |  |
| Everyday racism | 0.1151 | 0.4057*** | 1.0000 |  |  |
| Anxiety-related symptoms | -0.0242 | 0.1777* | 0.2786** | 1.0000 |  |
| Depressive symptoms | -0.1158 | 0.1513* | 0.3155*** | 0.7468*** | 1.0000 |

* p < 0.05, ** p < 0.01, *** p < 0.001
